# Progressive and self-limiting neurodegenerative disorders in Africa: a new prominent field of research led by South Africa but without strong health policy

**DOI:** 10.11604/pamj.2016.23.220.8475

**Published:** 2016-04-22

**Authors:** Brice Poreau

**Affiliations:** 1 CHU Grenoble Alpes, F-38000, Grenoble, France

**Keywords:** Neurodegenerative disorders, strong health policy, mortality, morbidity

## Abstract

**Introduction:**

Neurodegenerative disorders are involved in mortality and morbidity of every country. A high prevalence is estimated in Africa. Neurodegenerative disorders are defined by a progressive or self-limiting alteration of neurons implied in specific functional and anatomical functions. It encompasses a various range of clinical disorders from self-limiting to progressive. Focus on public health policies and scientific research is needed to understand the mechanisms to reduce this high prevalence. We use bibliometrics and mapping tools to explore the area studies and countries involved in scientific research on neurodegenerative disorders in Africa.

**Methods:**

We used two databases: Web of Science and Pubmed. We analyzed the journals, most cited articles, authors, publication years, organizations, funding agencies, countries and keywords in Web of Science Core collection database and publication years and Medical Subject Headings in Pubmed database. We mapped the data using VOSviewer.

**Results:**

We accessed 44 articles published between 1975 and 2014 in Web of Science Core collection Database and 669 from Pubmed database. The majority of which were after 2006. The main countries involved in research on neurodegenerative disorders in Africa the USA, the United Kingdom, France and South Africa representing the main network collaboration. Clinical neurology and Genetics hereditary are the main Web of Science categories whereas Neurosciences and Biochemistry and Molecular Biology are the main Web of Science categories for the general search “neurodegenerative disorders” not restrained to Africa. This is confirmed by Medical Subject Headings analysis from Pubmed with one more area study: Treatment.

**Conclusion:**

Neurodegenerative disorders research is leaded by South Africa with a network involving the USA, the UK, as well as African countries such Zambia. The chief field that emerged was on patient and hereditary as well as treatment. Public health policies were lacking fields in research whereas prevalence is estimated to be important in every country. New 17 sustainable development goals of the United Nations could help in this way.

## Introduction

Neurodegenerative disorders (ND) encompass various pathologies from well-identified genetic abnormalities such as Huntington's disease [1] to multifactorial mechanisms such as Parkinson disease or Alzheimer disease [2, 3]. ND are characterized by a progressive dysfunction of the central nervous system. Definitions of neurodegeneration can be multiple and we define it in this paper as a progressive or self-limiting alteration of neurons implied in specific functional and anatomical functions [4]. Nevertheless, clinical findings of ND are not only neurologic and can also be multisystemic, it means with dysfunctions of other organs such as in Huntington's disease [5]. Prevalence of neurodegenerative disorders is very high. For example, in Europe, only Parkinson disease is estimated from 65.6 per 100,000 to 12,500 per 100,000 inhabitants [6]. Costs are huge; estimation in 2004 in Europe is 386 billion euros [7]. Neurodegenerative disorders are involved in mortality and morbidity of every country. A high prevalence is estimated in Africa [8]. Focus on public health policies and scientific research is needed to understand the mechanisms to reduce this prevalence. In order to focus on a Africa-relevant approach, I will study both progressive and self-limiting neurodegenerative disorders, including, for example Nodding Syndrome (progressive) and cassavism, lathyrism (self-limiting). All of these disorders have been and are likely to continue to be endemic/epidemic regionally in Africa [9, 10]. The aim of this article is to analyze scientific publications on neurodegenerative disorders in Africa and to determine the links between the countries involved and the area studies.

## Methods

We used previously described methods [11, 12]. In brief, we accessed through two databases: the Science Citation Index-Expanded (SCIE) database Core collection, from the Web of Science (WOS) platform Thomson Reuters and Pubmed database. Concerning the WOS database, in the advanced search from WOS, we obtained the articles using this formula: TS=(Neurodegenerative disorders AND Africa) for the period 1975-2014. We verified each record to ensure its relevance. There were no restrictions regarding the document types. Then, we performed the “analysis results” function of WOS. We extracted: journals, most cited articles, authors, countries, funding agencies, organizations, publication years and Web of Science Categories. In order to evaluate the research networks between countries, after the analysis by country, we viewed the records of each country and then performed the analysis a second time in order to understand the links between the chosen country and the other countries. We then established the mapping diagram. To analyze the Web of Science Categories, we exported the date into a file “analyze.txt”. This file can be read by the program wc10.exe. It generated map-files for VOSviewer [12–14]. These analyses were to compare with the same search but without “Africa”. It means we performed the search TS = Neurodegenerative in WOS database. We added search of another database: Pubmed, to extend the results of the WOS database. We obtained publications using this formula: (Neurodegenerative disorders AND Africa) AND (“1900”(Date - Publication): “2014/12/31”(Date - Publication)). We extracted publication years. We extracted data with MEDLINE file. We then analyzed Medical Subject Headings (MeSH) with previously method described. We generated VOS-viewer picture as described [12–14]. The WOS core collection database and Pubmed database were used to perform our study. Publications from African countries could be not indexed in these databases.

## Results

**Figure 1:**
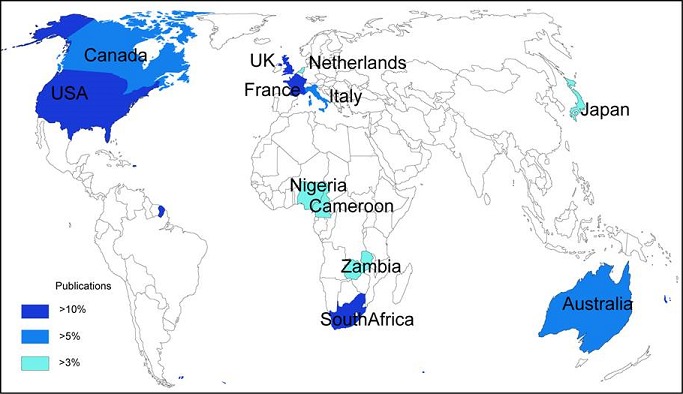
Countries involved in neurodegenerative disorders research in Africa according to the percentage of publications in WOS database. The United States of America (USA), the United Kingdom (UK), South Africa and France are the main countries

**Figure 2:**
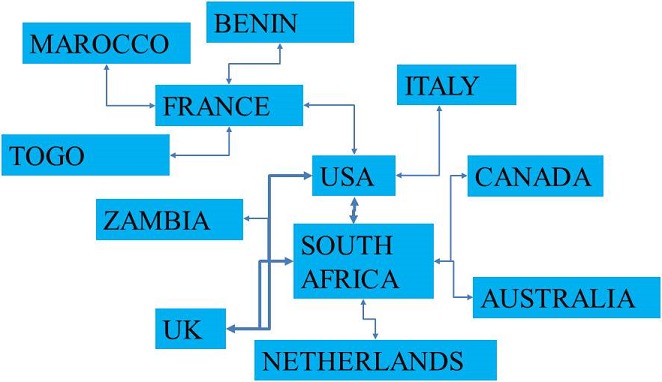
Publication collaboration networks between the countries involved in neurodegenerative disorders research in Africa, according to WOS database. The main network is between South Africa, US and UK

**Figure 3:**
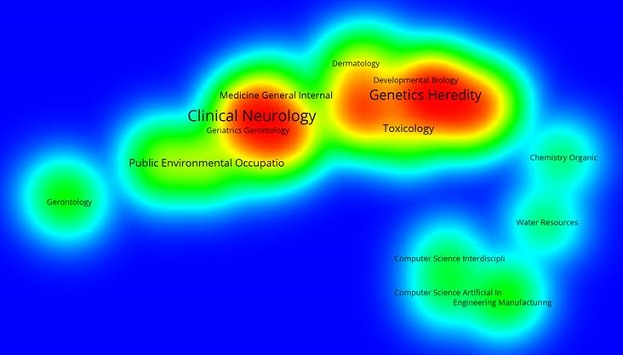
Web of Science categories for the search “Neurodegenerative disorders AND Africa”. The main categories are Clinical Neurology and Genetics Heredity

**Figure 4:**
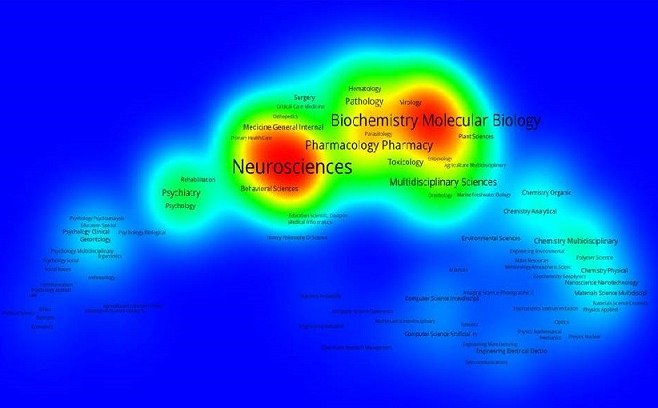
Web of Science categories for the search “Neurodegenerative disorders”. The main categories are Neurosciences and Biochemistry Molecular Biology

**Figure 5:**
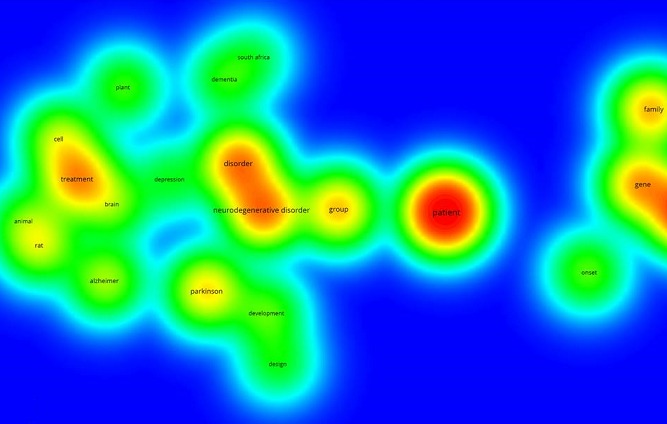
Medical Subject Headings (MeSH) of the search “Neurodegenerative disorders AND Africa” in Pubmed database. The main MeSH are patient, mutation, neurodegenerative disorder and treatment

Using WOS core collection database, we obtained 44 records. More than 68% of the records were after 2006 (Table 1). The four authors with most records were Van Staden (South Africa), Aderogba (South Africa), Krausen (South Africa) and Ndhlala (South Africa). The main journals were European Journal of Human Genetics (6,8%), Journal of Ethnopharmacology (6,8%), African Journal of Biotechnology (4,5%), Journal of the Neurological Sciences (4,5%), Movement disorders (4,5%), South African Medical Journal (4,5%). The main research funding agencies were the University of Kwazulu-Natal (South Africa) (6,8%), UK Parkinson disease Society (4,5%), The University of Cape Town Research Council (South Africa) (4,5%) and the National Research Fundation (South Africa) (4,5%). The four most represented institutions were the University of Cape-Town (11,3%), INSERM (French Institute of Health and Medical Research, France) (9%), the University of Kwazulu-Natal (South Africa) (9%), The University of Witwatersrand (South Africa) (9%). The main countries involved were South Africa (31,8%), the United States of America (29,5%), the United Kingdom (18,18%), France (11,3%), Australia (6,8%), Canada (6,8%) and Italy (6,8%). We presented the countries involved according to the percentage of publications found in WOS database (Figure 1). Then, we mapped the networks between the main countries (Figure 2). The scientific network concerning research on neurodegenerative disorders, with the main countries involved, is described. The main arrow/link is between the USA, the United Kingdom and South Africa as the most important collaboration. Others networks are involved with France, Canada, Japan, Italy, the Netherlands and African countries such as Zambia, Morocco, Togo and Benin. Finally, we used VOSviewer to map the Web of Science Categories (Figure 3). The most important categories were Clinical neurology and Genetics Heredity. We compare it to the map of the search “neurodegenerative disorders” without restraining to Africa (Figure 4). The most important categories were Neurosciences and Biochemistry Molecular Biology in that case, showing differences between publications specifically focused on Africa or on neurodegenerative disorders in general. We then compared the data from WOS database with another database: Pubmed. We extracted 669 publications. We presented the publication years and 38% were after 2006 (Figure 2). This result is concordant with WOS database. Moreover, more publications were found in percentage before 2006, and first publications were in the 1960s. We extracted the Medical Subject Headings (MeSH), and we mapped with VOSviewer (Figure 5). The main MeSH are patient, mutation, neurodegenerative disorder and treatment. The first MeSH are concordant with WOS categories (Figure 3): patient, mutation, and neurodegenerative disorder with Clinical neurology and Genetics Heredity. We also found the MeSH treatment in pubmed. This topic is not found with WOS database.

**Table 1:** Records (n = 44, WOS database) per year from 1975 to 2014 (68% of publications were after 2006)

Publication Years	records	Percentage (%)
2014	8	18,182
2013	4	9,091
2012	7	15,909
2011	2	4,545
2010	3	6,818
2009	3	6,818
2008	1	2,273
2007	1	2,273
2006	1	2,273
2005	1	2,273
2004	2	4,545
2000	1	2,273
1999	3	6,818
1998	3	6,818
1997	1	2,273
1995	3	6,818

## Discussion

The results of our analysis of two databases present a majority of publications after 2006 (Table 1 and Table 2). Pubmed database allow us to say that studies on neurodegenerative disorders in Africa were performed decades ago [15]. Neurodegenerative disorders are therefore a burning issue for the African continent.

**Table 2:** Records (n = 669, Pubmed database) per year from 1964 to 2015 (38% of publications were after 2006)

Publications years	Records	Percentage	Publications years	Records	Percentage
2014	62	9,012	1989	8	1,163
2013	5	0,727	1988	12	1,744
2012	52	7,558	1987	7	1,017
2011	41	5,959	1986	9	1,308
2010	4	0,581	1985	4	0,581
2009	4	0,581	1984	7	1,017
2008	42	6,105	1983	3	0,436
2007	17	2,471	1982	4	0,581
2006	34	4,942	1981	5	0,727
2005	19	2,762	1980	6	0,872
2004	26	3,779	1979	5	0,727
2003	28	4,070	1978	2	0,291
2002	22	3,198	1977	3	0,436
2001	24	3,488	1976	1	0,145
2000	21	3,052	1975	1	0,145
1999	26	3,779	1974	2	0,291
1998	15	2,180	1973	5	0,727
1997	22	3,198	1972	8	1,163
1996	18	2,616	1971	1	0,145
1995	17	2,471	1970	4	0,581
1994	16	2,326	1969	3	0,436
1993	16	2,326	1967	2	0,291
1992	15	2,180	1966	2	0,291
1991	11	1,599	1964	3	0,436
1990	5	0,727			

The increase of publications after 2006 is concordant with the general increase of publications in these two databases. Nevertheless the general volume of publications is very low. In fact, only with the example of WOS database, 44 publications were found with the search “neurodegenerative disorders AND Africa” whereas we found more than 24000 publications with the search “Neurodegenerative disorders” without restraining to Africa. It represents only 0, 18% of the publications of this fields of research. It means that the area studies are different if we restrain or not to Africa. According to Figure 2 and Figure 3, research in Africa appears to be focused on clinics. The goal is therefore to perform the diagnosis which seems under diagnosed [8; 16–17]. Moreover, different disease-specific hotspots can be determined.

To discuss these results, I will focus on the distinction between progressive and self-limiting disorders. Self-limiting neurological disorders can be defined as presence of neurological dysfunction with permanent or regressive state but without progression of the neurological alterations. The search “Neurodegenerative disorders” without Africa focuses on progressive disorders with for example Alzheimer's disease, Huntington's disease or Parkinson's disease. In the case of the search on Africa, a self-limiting disease-specific hotspot is emerging. The main example is about cassavism and lathyrism [10]. These diseases are caused by a high exposure to cassava roots for the first one and grass pea for the second one. Clinical features are not distinguishable between both diseases and consist in spastic paraparesis and crippling disability. This specific field of research on neurodegenerative disorders and Africa is described by a most prominent field of research in toxicology as well as the clinical neurology field (Figure 3). In these cases, prevention is therefore possible. The patients are known to be the poorest section of the population [10]. Education and fighting against illiteracy are necessary. Moreover, genetically modified crops could be used to avoid cassavism and lathyrism. Self-limiting neurological disorders are therefore an African specific hotspot.

Progressive disorders are also a main issue for Africa with also specific diseases. Three main examples of progressive disorders found in Africa and other continents can be developed: Parkinson's disease, Alzheimer's disease and Huntington's disease. The first one results in dopamine deficiency within the basal ganglia and leads to slowly progressive motor impairments. Management is possible but as the aetiology and pathogenesis are complex and not well understood, Parkinson's disease treatments are not fully sufficient [2]. Concerning Alzheimer's disease, it is due to amyloid plaques leading to dementia. As the first one, the aetiology and pathogenesis remain unclear, therefore management of patient is very difficult [3]. For the third one, Huntington's disease, it is caused by a polyglutamine stretch in the gene Huntingtin. The clearly genetic abnormality results in the expression of less normal protein and the production of a mutant protein. Clinical features are chorea, motor and psychiatric impairments and it occurs as the age of 30-35 years old. It is a progressive disorder leading to death. There is no treatment [1]. For these examples of progressive neurological disorders, African countries propose the management of the patients [16, 17]. It means that a structure is needed with physicians trained for diagnosis, predictive genetic testing in the case of some neurodegenerative disorders as Huntington's disease, and follow-up. South Africa is the main country involved in such action [18]. That's why we found in our analysis that the main institutions and the main founding agencies were South African. Nevertheless, number of structures and physicians remain not sufficient. The World Health Organization notes that Africa has the lowest median number of neurological beds per 10 000 population and the lowest number of neurologists per 100000 population [19]. Therefore, in the fight against progressive neurological disorders, collaboration and networks are needed. We didn't find for example a main network between African countries (Figure 2). This last point is problematic as in the case of progressive disorders, some diseases appear to be endemic to Africa as Nodding syndrome [9]. Patients are found in Uganda, Tanzania, Liberia and Sudan. Clinical signs consist in involuntary dropping of the head. Other signs as seizure are associated. No aetiology has been yet known. There is no treatment.

If a main African collaboration network is needed, international networks are also necessary. The USA and the UK, as well as France are present in both fields of research on self-limiting and progressive neurological disorders. But, we already present that neurodegenerative disorders are not, for instance, one of the main public health policies in Africa except for HIV-related progressive neurological disorders [10, 11]. Millennium development goals were not focused on neurodegenerative disorders. HIV, malaria, tuberculosis are clearly mentioned and the policies on fighting these diseases were funded but progressive neurodegenerative disorders such as Parkinson, Alzheimer and other diseases and self-limiting neurological disorders are not mentioned [20].

The United Nations Sustainable Development Summit on 25 September 2015 proposed 17 sustainable development goals. The goal number 3 is entitled “Good health and well-being”. Therefore, neurodegenerative disorders are clearly involved in this topic, as a non-communicable disease. The African continent with a huge prevalence of neurodegenerative disorders [8] could benefit from the experiences of networks on research, therapeutic trials, follow-up, structures, patients organization like associations, funding of other countries.

## Conclusion

In conclusion, neurodegenerative disorders are a main burning issue for the future of Africa. South Africa remains the leader in this field. Nevertheless, an increase of structures, physicians, collaborations is needed with a clear public health policy focused on neurodegeneration.

### What is known about this topic

- Neurodegenerative disorders are involved in mortality and morbidity of every country;
- High prevalence of neurodegenerative disorders is estimated in Africa;
- Both progressive and self-limiting neurodegenerative disorders are present in Africa.

### What this study adds

- Neurodegenerative disorders research is leaded by South Africa with a network involving the USA, the UK, as well as African countries such Zambia;
- The chief field that emerged was on patient and hereditary as well as treatment;
- Public health policies were lacking fields in research.
